# Supplementary material for: Integrative mRNA and microRNA Analysis Exploring the Inducing Effect and Mechanism of Diallyl Trisulfide (DATS) on Potato against Late Blight
Source: Int J Mol Sci. 2023 Feb 9;24(4):3474. doi: 10.3390/ijms24043474 (PMC9962630; doi:10.3390/ijms24043474)
Supplement: Supplementary file 1 [file ijms-24-03474-s001.zip › Supplementary Table S1.pdf]

Supplementary Table S1 Classification criteria for potato late blight disease

Disease classification	Grading standard (in plants)
Level 0	No disease, the whole leaves is intact
Level 1	Only few leaves display lesions
Level 3	The leaf area of the lesions accounts for 20 – 40 % of the total leaf area
Level 5	Leaf area with lesions accounts for 41 – 60 % of the total leaf area
Level 7	Leaf area with lesions accounts for 61 – 80 % of the total leaf area
Level 9	The leaf area of the lesions accounts for more than 81 % of the total leaf area or total necrosis
