# Supplementary material for: Integrative mRNA and microRNA Analysis Exploring the Inducing Effect and Mechanism of Diallyl Trisulfide (DATS) on Potato against Late Blight
Source: Int J Mol Sci. 2023 Feb 9;24(4):3474. doi: 10.3390/ijms24043474 (PMC9962630; doi:10.3390/ijms24043474)
Supplement: Supplementary file 1 [file ijms-24-03474-s001.zip › Supplementary Table S2.pdf]

Supplementary Table S2 Information about primer sequences used in this study

Gene name (or ID)	Primer	Sequence (5'-3')	Reference
Stef1α	F EF1a RT	ATTGGAAACGGATATGCTCCA	(Zhou et al., 2019)
	R EF1a RT	TCCTTACCTGAACGCCTGTCA	
StActin	F StActin RT	GACAACGGAACTGCACGATC	(Li et al., 2019)
	R StActin RT	TACGCTGAGCTTCATCACCA	
PGSC0003DMG400032829	F StC3HLF RT	GCTGATCCGTACTCGTCT	This study
	R StC3HLF RT	GCCAACACTCAAACACCC	
PGSC0003DMG400005657	F StMYB-like RT	ACAATCAGTGTCGCAGGAG	This study
	R StMYB-like RT	TGGGTCTAAGGGCAGGT	
PGSC0003DMG400018397	F StWIP RT	GAGCGTGGATAGTAGCAG	This study
	R StWIP RT	CAATTTGGACCCCAACAG	
PGSC0003DMG400006643	F StWI12 RT	CTCTGGAGGAAACAACAA	This study
	R StWI12RT	AACAACGGAACCGAATA	
PGSC0003DMG400018373	F StWIP(h) RT	CCGTCGCCGTCGTAAACA	This study
	R StWIP(h) RT	TTCCGCCTCCGATGAACCC	
