# Supplementary material for: Integrative mRNA and microRNA Analysis Exploring the Inducing Effect and Mechanism of Diallyl Trisulfide (DATS) on Potato against Late Blight
Source: Int J Mol Sci. 2023 Feb 9;24(4):3474. doi: 10.3390/ijms24043474 (PMC9962630; doi:10.3390/ijms24043474)
Supplement: Supplementary file 1 [file ijms-24-03474-s001.zip › Supplementary Table S3.pdf]

Supplementary Table S3 Summary of transcriptomics sequencing data

**Samples**	**Clean reads**	**Clean bases**	**GC Content**	**Q30 (%)**
DATS1	24,200,437	7,246,582,050	43.60 %	93.93 %
DATS2	22,710,420	6,798,874,264	43.41 %	94.21 %
DATS3	23,117,152	6,916,884,684	43.55 %	93.89 %
CK1	23,993,991	7,181,418,566	43.70 %	93.34 %
CK2	26,309,436	7,871,672,092	43.25 %	94.09 %
CK3	23,301,649	6,972,468,148	43.25 %	92.75 %
