# Supplementary material for: Integrative mRNA and microRNA Analysis Exploring the Inducing Effect and Mechanism of Diallyl Trisulfide (DATS) on Potato against Late Blight
Source: Int J Mol Sci. 2023 Feb 9;24(4):3474. doi: 10.3390/ijms24043474 (PMC9962630; doi:10.3390/ijms24043474)
Supplement: Supplementary file 1 [file ijms-24-03474-s001.zip › Supplementary Table S5.pdf]

Supplementary Table S5 Gene Ontology enrichment analysis of DEGs ($P < 0.05$).

Category	GO.ID	Term	Number of genes	Number of DEGs	p Value
MF	GO:0015035	protein disulfide oxidoreductase activity	102	3	2.80E-07
	GO:0020037	heme binding	677	14	7.80E-07
	GO:0031409	pigment binding	35	1	2.40E-06
	GO:0016168	chlorophyll binding	52	1	2.70E-05
	GO:0004867	serine-type endopeptidase inhibitor activity	31	2	0.00012
	GO:0016709	oxidoreductase activity	356	9	0.00013
	GO:0004866	endopeptidase inhibitor activity	83	7	0.0002
	GO:0010329	auxin efflux transmembrane transporter activity	14	1	0.0011
	GO:0003700	transcription factor activity, sequence-specific DNA binding	703	15	0.00212
	GO:0046943	carboxylic acid transmembrane transporter activity	105	4	0.00259
CC	GO:0016021	integral component of membrane	6364	133	3.30E-07
	GO:0009522	photosystem I	56	1	3.40E-05
	GO:0009523	photosystem II	71	1	0.00011
	GO:0005576	extracellular region	548	7	0.00107
	GO:0005773	vacuole	426	16	0.00144
	GO:0010287	plastoglobule	52	1	0.00236
	GO:0031225	anchored component of membrane	144	7	0.00517
	GO:0009941	chloroplast envelope	273	8	0.00802
	GO:0071944	cell periphery	1767	41	0.00833
	GO:0009505	plant-type cell wall	136	3	0.00887
BP	GO:0010951	negative regulation of endopeptidase activity	82	7	3.60E-09
	GO:0009733	response to auxin	285	6	6.20E-09
	GO:0045454	cell redox homeostasis	142	4	1.40E-06
	GO:0009768	photosynthesis, light harvesting in photosystem I	35	1	2.80E-06
	GO:0006355	regulation of transcription, DNA-templated	1592	29	2.50E-05
	GO:0018298	protein-chromophore linkage	54	1	9.60E-05
	GO:0006457	protein folding	197	4	0.0003
	GO:0009926	auxin polar transport	70	2	0.00041
	GO:0042744	hydrogen peroxide catabolic process	117	1	0.0005
	GO:0098656	anion transmembrane transport	151	4	0.00124
