# Supplementary material for: Integrative mRNA and microRNA Analysis Exploring the Inducing Effect and Mechanism of Diallyl Trisulfide (DATS) on Potato against Late Blight
Source: Int J Mol Sci. 2023 Feb 9;24(4):3474. doi: 10.3390/ijms24043474 (PMC9962630; doi:10.3390/ijms24043474)
Supplement: Supplementary file 1 [file ijms-24-03474-s001.zip › Supplementary Table S6.pdf]

Supplementary Table S6 KEGG pathway annotations results.

#Pathway	ko_ID	DEG	Gene
2-Oxocarboxylic acid metabolism	ko01210	2	73
Linoleic acid metabolism	ko00591	1	28
Fatty acid degradation	ko00071	2	60
RNA polymerase	ko03020	1	63
Ribosome	ko03010	1	378
Tyrosine metabolism	ko00350	2	74
RNA transport	ko03013	2	213
Phenylalanine, tyrosine and tryptophan biosynthesis	ko00400	1	60
Tropane, piperidine and pyridine alkaloid biosynthesis	ko00960	1	44
Carbon fixation in photosynthetic organisms	ko00710	2	86
Basal transcription factors	ko03022	2	49
Fatty acid metabolism	ko01212	1	113
Histidine metabolism	ko00340	1	42
Glycerolipid metabolism	ko00561	1	89
Phagosome	ko04145	1	104
Nucleotide excision repair	ko03420	2	125
Fatty acid elongation	ko00062	1	44
Cysteine and methionine metabolism	ko00270	1	133
Citrate cycle (TCA cycle)	ko00020	1	66
Ubiquitin mediated proteolysis	ko04120	1	210
Ubiquinone and other terpenoid-quinone biosynthesis	ko00130	3	46
Phosphatidylinositol signaling system	ko04070	1	91
Oxidative phosphorylation	ko00190	1	176
Glutathione metabolism	ko00480	3	129
Mismatch repair	ko03430	1	109
Alanine, aspartate and glutamate metabolism	ko00250	2	58
Peroxisome	ko04146	3	118
Phenylpropanoid biosynthesis	ko00940	9	254
ABC transporters	ko02010	1	33
Glucosinolate biosynthesis	ko00966	2	14
Glycine, serine and threonine metabolism	ko00260	1	87
Glyoxylate and dicarboxylate metabolism	ko00630	3	90
Valine, leucine and isoleucine degradation	ko00280	1	88
Inositol phosphate metabolism	ko00562	2	74
Synthesis and degradation of ketone bodies	ko00072	1	10
Starch and sucrose metabolism	ko00500	12	285
Protein processing in endoplasmic reticulum	ko04141	5	287
Plant hormone signal transduction	ko04075	11	400
Pentose phosphate pathway	ko00030	2	64
Photosynthesis - antenna proteins	ko00196	1	38
alpha-Linolenic acid metabolism	ko00592	3	60
Cyanoamino acid metabolism	ko00460	3	65

beta-Alanine metabolism	ko00410	2	84
RNA degradation	ko03018	1	141
Biosynthesis of amino acids	ko01230	3	270
Nitrogen metabolism	ko00910	3	40
Thiamine metabolism	ko00730	1	14
Butanoate metabolism	ko00650	3	30
Flavonoid biosynthesis	ko00941	3	66
Proteasome	ko03050	1	71
Isoquinoline alkaloid biosynthesis	ko00950	1	50
Taurine and hypotaurine metabolism	ko00430	4	16
Circadian rhythm - plant	ko04712	1	55
Tryptophan metabolism	ko00380	3	75
Ribosome biogenesis in eukaryotes	ko03008	1	118
mRNA surveillance pathway	ko03015	1	151
Glycolysis / Gluconeogenesis	ko00010	4	150
Base excision repair	ko03410	1	60
Purine metabolism	ko00230	3	192
Porphyrin and chlorophyll metabolism	ko00860	4	53
Pyruvate metabolism	ko00620	2	110
SNARE interactions in vesicular transport	ko04130	1	47
Fructose and mannose metabolism	ko00051	1	79
Arginine and proline metabolism	ko00330	1	73
Phenylalanine metabolism	ko00360	4	66
Photosynthesis	ko00195	1	83
Spliceosome	ko03040	1	234
Terpenoid backbone biosynthesis	ko00900	4	80
Endocytosis	ko04144	1	238
Galactose metabolism	ko00052	4	69
Sesquiterpenoid and triterpenoid biosynthesis	ko00909	1	31
Steroid biosynthesis	ko00100	3	43
Cutin, suberine and wax biosynthesis	ko00073	3	43
AGE-RAGE signaling pathway in diabetic complications	ko04933	1	21
Plant-pathogen interaction	ko04626	4	247
Pyrimidine metabolism	ko00240	1	149
Stilbenoid, diarylheptanoid and gingerol biosynthesis	ko00945	3	48
Amino sugar and nucleotide sugar metabolism	ko00520	5	167
Zeatin biosynthesis	ko00908	1	153
Carbon metabolism	ko01200	7	323
