# Supplementary material for: Integrative mRNA and microRNA Analysis Exploring the Inducing Effect and Mechanism of Diallyl Trisulfide (DATS) on Potato against Late Blight
Source: Int J Mol Sci. 2023 Feb 9;24(4):3474. doi: 10.3390/ijms24043474 (PMC9962630; doi:10.3390/ijms24043474)
Supplement: Supplementary file 1 [file ijms-24-03474-s001.zip › Supplementary Table S7.pdf]

Supplementary Table S7 Summary of miRNA sequencing data

Samples	Raw_reads	Low_quality	Containing'N'reads	Length < 18	Length > 30	Clean_reads	Q30 (%)
DATS1	17,672,060	0	0	848,390	4,839,451	11,984,219	96.67
DATS2	15,298,512	0	0	4,378,201	933,995	9,986,316	96.8
DATS3	28,546,683	0	0	1,910,291	10,369,800	16,266,592	96.58
CK1	30,045,187	0	0	5,988,070	5,057,789	18,999,328	96.77
CK2	16,038,412	0	0	2,212,689	1,460,391	12,365,332	96.74
CK3	15,050,022	0	0	2,071,958	1,377,035	11,601,029	96.77
