# Supplementary material for: Integrative mRNA and microRNA Analysis Exploring the Inducing Effect and Mechanism of Diallyl Trisulfide (DATS) on Potato against Late Blight
Source: Int J Mol Sci. 2023 Feb 9;24(4):3474. doi: 10.3390/ijms24043474 (PMC9962630; doi:10.3390/ijms24043474)
Supplement: Supplementary file 1 [file ijms-24-03474-s001.zip › Supplementary Table S8.pdf]

Supplementary Table S8 Summary of miRNA results for each sample

Samples	Known-miRNAs	Novel-miRNAs	Total
DATS1	140	296	436
DATS2	96	283	379
DATS3	140	295	435
CK1	111	286	397
CK2	120	294	414
CK3	101	282	383
Total	155	296	451
